# A multiscale theory for spreading and migration of adhesion-reinforced mesenchymal cells

**DOI:** 10.1098/rsif.2023.0317

**Published:** 2023-12-13

**Authors:** Wenya Shu, C. Nadir Kaplan

**Affiliations:** ^1^ Department of Physics, Virginia Polytechnic Institute and State University, Blacksburg, VA 24061, USA; ^2^ Center for Soft Matter and Biological Physics, Virginia Polytechnic Institute and State University, Blacksburg, VA 24061, USA

**Keywords:** mechanobiology, mesenchymal migration, adhesion reinforcement, durotaxis, viscotaxis

## Abstract

We present a chemomechanical whole-cell theory for the spreading and migration dynamics of mesenchymal cells that can actively reinforce their adhesion to an underlying viscoelastic substrate as a function of its stiffness. Our multiscale model couples the adhesion reinforcement effect at the subcellular scale with the nonlinear mechanics of the nucleus–cytoskeletal network complex at the cellular scale to explain the concurrent monotonic area–stiffness and non-monotonic speed–stiffness relationships observed in experiments: we consider that large cell spreading on stiff substrates flattens the nucleus, increasing the viscous drag force on it. The resulting force balance dictates a reduction in the migration speed on stiff substrates. We also reproduce the experimental influence of the substrate viscosity on the cell spreading area and migration speed by elucidating how the viscosity may either maintain adhesion reinforcement or prevent it depending on the substrate stiffness. Additionally, our model captures the experimental directed migration behaviour of the adhesion-reinforced cells along a stiffness gradient, known as durotaxis, as well as up or down a viscosity gradient (viscotaxis or anti-viscotaxis), the cell moving towards an optimal viscosity in either case. Overall, our theory explains the intertwined mechanics of the cell spreading, migration speed and direction in the presence of the molecular adhesion reinforcement mechanism.

## 1. Introduction

Cell motion plays a crucial role in various biological processes ranging from tissue formation to metastasis. The mechanical properties of the extracellular matrix (ECM) are critical environmental cues that orchestrate the spreading and migration of adherent (mesenchymal) cells [1–8]. Focal adhesion (FA) sites play a key role in cell mechanosensitivity by mediating the chemical and mechanical interactions between the cell and ECM. The FA dynamics can directly impact the chemically driven localized protrusions and contractions across the cell, governed by the tightly coupled spatio-temporal distributions of the proteins, such as the Rho GTPases, ROCK or CDC42, which influence the migration dynamics [9–14]. Thus, a rigorous mathematical description of cell migration must include accurate modelling of the adhesion forces at different FA sites and their coupling with the cytoskeletal dynamics driven by the intracellular signalling pathways.

Although some cells such as the U-251MG glioblastoma cells exhibit a biphasic dependence of the traction force on the rigidity of the underlying matrix [15,16], which can be explained by the classical motor-clutch theory [17,18], many other systems, such as endothelial cells and fibroblasts, display a monotonic rigidity–force relationship [19,20]. This behaviour is attributed to active adhesion reinforcement triggered by the unfolding of the talin molecules that leads to the vinculin binding and in turn to an increased integrin density at an FA site [21,22]. While an augmented motor-clutch theory with adhesion reinforcement successfully captures the monotonic increase of the traction force with the matrix stiffness [23], it cannot explain the non-monotonic dependence of the migration speed on the ECM rigidity measured in the experiments [5–8]. This suggests that the mutual dynamics of the many FA sites across the cell along with the cytoskeletal dynamics must be considered to relate the migration speed to the traction forces and in turn to the matrix rigidity, rather than at a single FA site. To reproduce the cell migration dynamics as a coordinated sequence of peripheral protrusion and contractions [4,24,25], whole-cell level formulations with multiple FA sites have been developed, including our recent theory that integrates the chemomechanical coupling between the Rho GTPase concentrations, the FA sites, the cytoskeletal network and the nucleus dynamics [26–28]. However, none of these studies have incorporated the adhesion reinforcement mechanism into the whole-cell dynamics. Importantly, we anticipate that a mere addition of the adhesion reinforcement effect to the whole-cell-scale model would still lead to a monotonic rigidity–speed relationship, implying further subtleties associated with the collective intracellular dynamics under adhesion reinforcement.

Here we generalize our multiscale theory in Shu & Kaplan [28] to elucidate the spreading and migration dynamics of adhesion-reinforced cells on viscoelastic substrates, overcoming the limitations of our previous model without adhesion reinforcement. Our model demonstrates how the nonlinear mechanics of the nucleus–cytoskeletal network complex must play a critical role in the experimental spreading and non-monotonic stiffness–speed profiles: large cell spreading on stiff substrates induced by the augmented local traction forces due to adhesion reinforcement, an effect accurately captured by our model, deforms the nucleus [29] and increases both the cytoplasmic viscosity and cytoskeletal stiffness [8,30–32]. Consequently, the drag force on the nucleus increases, making it harder for the cell to translocate the nucleus on stiff substrates, as evidenced by a theoretical non-monotonic stiffness–speed relationship in quantitative agreement with experiments. By using the standard linear solid (SLS) model for the substrate viscoelasticity, we also investigate the effect of the substrate stress relaxation on cell migration across a broad stiffness range, which is experimentally well documented [23,33–35]. We show that, on a substrate with high elastic stiffness, fast stress relaxation (low viscosity) may pre-empt the effect of adhesion reinforcement whereas slow relaxation (high viscosity) may promote it. That way, our model quantitatively reproduces the experimental spreading area and migration speed profiles of the HT-1080 human fibrosarcoma cells [27]. Furthermore, we demonstrate that adhesion-reinforced cells persistently move up the stiffness gradients, i.e. durotaxis [36–38], in good agreement with the experimental migration patterns of the MDA-MB-231 cells [16]. We also reveal the effect of the viscosity gradients on directed migration, a.k.a. viscotaxis, which sheds light on the migration of human mesenchymal stem cells from high to low loss moduli on collagen-coated polyacrylamide gels [39].

Our theory provides a rigorous understanding of how the nucleus drag as a function of cell spreading gives rise to the experimentally observed complex migration dynamics, in contrast with the previous phenomenological treatments that assumed a direct functional relationship between the drag force, the traction force and substrate stiffness [40,41]. Furthermore, because our model takes into account additional intracellular components beyond just the FA sites, it provides a plausible mechanism for the emergent non-uniform stiffness–speed relation that complements the cell model which relates cell speed only to the resultant traction force from the collective adhesion-reinforced FA dynamics [42].

## 2. Methods

Mesenchymal migration entails the spatio-temporal coordination of front protrusion driven by actin polymerization and focal adhesion (FA) followed by rear contraction due to focal de-adhesion and actomyosin activity. The front–rear symmetry is primarily broken by the chemical polarization of the active and inactive Rho GTPase proteins, which govern the dynamics of the FA sites.

**Figure 1.**
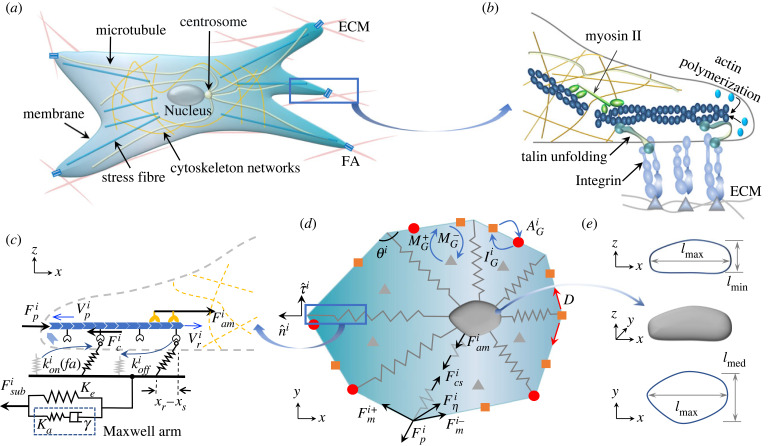
Multiscale whole-cell theory schematics. (*a*) Intracellular organization involved in mesenchymal migration. (*b*) Close-up view of the cytoskeleton–ECM linkage via integrins and the adaptor proteins (e.g. talin). (*c*) The parameters and variables of the motor-clutch model with adhesion reinforcement at each FA site. (*d*) The vertex-based model that couples the cell–matrix interactions at the subcellular scale to the chemomechanical dynamics at the cellular scale. The Rac1 concentration *R* and RhoA concentration *ρ* can each be in a membrane-bound active (red dots) or inactive state (orange squares), or dissolved in the cytoplasm (grey triangles). The conversion rates between these states are $A_{G}^{i}$, $I_{G}^{i}$, $M_{G}^{-}$, $M_{G}^{+}$ (*G* ≡ {*R*, *ρ*}); *D* is the diffusion constant of the membrane-bound species (equation (2.15)). The angle between the two membrane sections at a vertex is denoted by *θ*^*i*^. (*e*) The principal dimensions *l*_max_, *l*_med_, *l*_min_ of a deformed nucleus.

Here we generalize our previous multiscale framework for the talin-low cell migration, which accounted for this complexity by considering the feedback between the biological components in figure 1*a*, by including the effect of focal adhesion reinforcement due to talin unfolding (figure 1*b*) [28]. Our modified multiscale model takes into account the nonlinear strain-stiffening of the cytoskeleton and the augmented viscous drag force on the deformed cell nucleus under cell flattening, as well as uses the SLS model for the ECM viscoelasticity. At the subcellular scale, a motor-clutch model with adhesion reinforcement is employed to determine the effect of substrate rigidity on traction forces at each FA site (figure 1*c*). Since cells operate in low Reynolds numbers, we only consider the viscous forces arising from the nucleus–cytoplasm and cell membrane–substrate frictions at the cellular scale. In our model, the forces across the two scales are transmitted by the cytoskeleton, and the augmented viscous drag force on the deformed cell nucleus in turn determines the active motion of the cell (figure 1*d*,*e*).

### 2.1. Equations of motion at subcellular scale

We first present the calculation of the cell vertex displacements due to the FA site dynamics and the mechanical balance at the corresponding vertex. For a vertex *i* with position vector **x**^*i*^(*t*), the local spreading velocity $V_{s}^{i}\equiv {\dot{x}}^{i}$ needs to be determined at every time step. In our model, the radial component $V_{s,n}^{i}\equiv V_{s}^{i}\cdot {\hat{n}}^{i}$ is controlled by the active processes, i.e. actin polymerization and actomyosin contraction regulated by the Rho GTPase proteins, while the polar spreading speed $V_{s,\tau }^{i}\equiv V_{s}^{i}\cdot {\hat{\tau }}^{i}$ is set by the balance between the polar components of the passive forces due to the cell deformations (${\hat{n}}^{i},{\hat{\tau }}^{i}$ defined in figure 1*d*).

The active radial spreading of a vertex with a speed $V_{s,n}^{i}$ is fuelled mainly by F-actin formation with a polymerization speed $V_{\;p}^{i}$ and counteracted by the retrograde G-actin flow with a speed $V_{r}^{i}$, which yields

2.1 $$V_{s,n}^{i}=V_{\;p}^{i}-V_{r}^{i}.$$

The polymerization rate at the vertex *i* is given by the ratio of the active Rac1 concentration $R_{a}^{i}$ to its mean $\left\langle R_{a}^{i}\right\rangle$ averaged over all vertices as $V_{\;p}^{i}\equiv (R_{a}^{i}/\left\langle R_{a}^{i}\right\rangle )V_{\;p}^{0}$, where $V_{\;p}^{0}$ is the reference polymerization speed. The retrograde actin flow speed $V_{r}^{i}$ is promoted by the net resistance force against protrusion $F_{\;p}^{i}$ due to the cell membrane elasticity and myosin contractions (figure 1*b*,*c*) and impeded by an elastic restoring force $F_{c}^{i}$ due to the formation of molecular bonds by proteins such as integrins, talin and vinculin between F-actin and ECM. We thus propose a phenomenological relation

2.2 $$V_{r}^{i}=V_{0}\left(1-\frac{F_{\mathrm{am}}^{i}}{N_{m}^{i}f_{m}}\right),\;F_{\mathrm{am}}^{i}\equiv F_{c}^{i}-F_{\;p}^{i},$$

where *V*_0_ is the unloaded myosin motor speed, $N_{m}^{i}$ is the number of active myosin motors, and *f*_*m*_ is the force that stalls the activity of one myosin motor. Owing to the increased iteration requirements for calculating $V_{r}^{i}$ when reaching its minimum during loading–unloading focal adhesion cycles, we enforce the condition $V_{r}=\max(V_{r},0)$ that preserves the accuracy while reducing the computational load (see electronic supplementary material, §S1 and figure S1, S2). RhoA is known to induce myosin motor activation, leading to stress fibre formation and contractility [43,44]. Therefore, we assume that the myosin motor number is controlled by the active RhoA concentration $\rho_{a}^{i}$ and its mean $\left\langle \rho_{a}^{i}\right\rangle$ averaged over all vertices as $N_{m}^{i}(t)\equiv (\rho_{a}^{i}/\left\langle \rho_{a}^{i}\right\rangle )N_{m}^{0}$, where $N_{m}^{0}$ is the reference myosin motor number. Note that this assumption guarantees the constancy of the total motor number in the cell, in accordance with the conservation of mass [16,26]. In equation (2.2), the elastic restoring force $F_{c}^{i}$ is determined from the FA dynamics with adhesion reinforcement, which is triggered by the stiffness of the viscoelastic substrate. On the other hand, the protrusion force $F_{\;p}^{i}$ is set by the local force balance at each vertex in the presence of the nonlinear strain stiffening of the cytoskeleton. To calculate these two force strengths, we detail each of those processes next.

#### 2.1.1. Focal adhesion dynamics with adhesion reinforcement

To account for the adhesion reinforcement due to talin unfolding, we extend the augmented motor-clutch model for FA dynamics introduced by Elosegui-Artola *et al*. [21] and Gong *et al*. [23] in order to calculate the clutch force $F_{c}^{i}$ at each vertex. By denoting average displacements of all bounded clutches at the filament end by $x_{r}^{i}(t)$ and the displacement of the substrate by $x_{\mathrm{sub}}^{i}$, the engaged clutch is represented by a Hookean spring with tension $f_{c}^{i}=K_{c}(x_{r}^{i}-x_{\mathrm{sub}}^{i})$ (*K*_*c*_: spring stiffness) [45,46]. The clutch force $f_{c}^{i}$ displays oscillatory dynamics driven by the cyclic process of binding, loading and unbinding within the system (electronic supplementary material, figure S3). At any instant *t*, the unbounded clutches associate with a rate $k_{\mathrm{on}}^{i}$. For the talin-low cells, a constant association rate $k_{\mathrm{on}}^{0}$ is typically assumed, corresponding to a constant clutch binding timescale $\tau_{\mathrm{on}}^{0}\equiv 1/k_{\mathrm{on}}^{0}$ [17,23,28]. However, a cell can activate adhesion reinforcement once it probes the elevated loading rate and high force per clutch before talin unfolding takes place. We thus introduce the adhesion reinforcement by assuming that, when the time-averaged clutch force ${\left\langle f_{a}^{i}\right\rangle }_{\tau_{l}}\equiv \int_{0}^{\tau_{l}}{\tilde{\;f}}_{c}^{i}\;d\tilde{t}/\tau_{l}$ (*τ*_*l*_: variable focal adhesion lifetime) without adhesion reinforcement is above a threshold force *f*_cr_, the clutch binding rate will increase per [21,23],

2.3 $$k_{\mathrm{on}}^{i}=k_{\mathrm{on}}^{0}\left(1+e^{\zeta ({\left\langle f_{a}^{i}\right\rangle }_{\tau_{l}}-f_{\mathrm{cr}})}\right),$$

where *ζ* is a characteristic inverse force scale. Since ${\left\langle f_{a}^{i}\right\rangle }_{\tau_{l}}$ must be obtained under the assumption that the adhesion reinforcement be absent, we separately calculate it by solving an isolated motor-clutch system with *ζ* = 0 (equations (2.2)–(2.6), see inset in electronic supplementary material, figure S4 and §S2), considering the updated protrusion force $F_{\;p}^{i}$ and the updated number of clutches $N_{c}^{i}$ and motors $N_{m}^{i}$ at vertex *i*. The clutch force ${\tilde{\;f}}_{c}^{i}$ and the time $\tilde{t}$ are variables in isolated motor-clutch modelling, distinct from the variables in our whole cell modelling. Owing to the time dependence of $F_{\;p}^{i},N_{c}^{i}$ and $N_{m}^{i}$, the variable ${\left\langle f_{a}^{i}\right\rangle }_{\tau_{l}}$ at every vertex must be updated at every *N* time steps. Our simulations have demonstrated that using *N* = 4000 time steps provides accurate predictions on cell migration speeds on stiff substrates, comparable to results obtained from more frequent updates of ${\left\langle f_{a}^{i}\right\rangle }_{\tau_{l}}$ (see electronic supplementary material, figure S5), while keeping computational costs reasonable.

Once formed, the molecular clutches must unbind at a dissociation rate $k_{\mathrm{off}}^{i}$ that depends on the clutch tension $f_{c}^{i}$. To that end, we use the functional form $k_{\mathrm{off}}^{i}\equiv k_{r0}\exp\left(f_{c}^{i}/f_{r0}\right)\;+k_{c0}\exp\left(-f_{c}^{i}/f_{c0}\right)$. Here, *k*_*r*0_ and *k*_*c*0_ denote the unloaded off-rate and the unloaded catch-rate, respectively, *f*_*r*0_ is the characteristic rupture force, and *f*_*c*0_ is the characteristic catch force. It follows that the fraction of the engaged clutches (0 ≤ *P*^*i*^(*t*) ≤ 1) is governed by the mean-field rate equation [23,45,46],

2.4 $$\frac{dP^{i}}{dt}=k_{\mathrm{on}}^{i}(1-P^{i})-k_{\mathrm{off}}^{i}P^{i}.$$

Denoting the number of available clutches as $N_{c}^{i}$, the total clutch force at the vertex *i* is then given by $F_{c}^{i}=P^{i}N_{c}^{i}f_{c}^{i}$. Here we incorporate the critical role of the Rac1 proteins in focal complex assembly by relating $N_{c}^{i}$ to the local Rac1 concentration, i.e. $N_{c}^{i}(t)\equiv (R_{a}^{i}/\left\langle R_{a}^{i}\right\rangle )N_{c}^{0}$, where $N_{c}^{0}$ denotes the reference clutch number [47–49]. This assumption implies that the total count of clutches $\sum_{i=1}^{N}N_{c}^{i}$ remains constant at any given moment, thereby adhering to the conservation of mass [16,26]. The mechanical equilibrium condition at the cell–substrate interface demands that the total force sustained by the engaged clutches must be balanced by the substrate deformation, leading to

2.5 $$F_{\mathrm{sub}}^{i}=F_{c}^{i}=P^{i}N_{c}^{i}K_{c}(x_{r}^{i}-x_{\mathrm{sub}}^{i}).$$

In equation (2.5), the substrate displacement $x_{\mathrm{sub}}^{i}$ is an unknown to be determined from a constitutive model for the substrate viscoelasticity, which we focus on next.

#### 2.1.2. Constitutive model for substrate viscoelasticity

Our previous implementation used the classical Kelvin–Voigt model for the substrate relaxation dynamics [28]. However, since the Kelvin–Voigt model predicts a very rigid non-physical behaviour when *t* < *τ*_*r*_ (*τ*_*r*_: substrate relaxation timescale) [50], it can result in a premature adhesion reinforcement. Comparatively, the SLS model allows for a better physical modelling by including a Maxwell arm (figure 1) [51]. It has also been demonstrated that the SLS model can capture the prominent relaxation timescale of the viscoelastic substrates fabricated by combining covalent and supramolecular cross-linking [23]. The SLS model expresses the constitutive relationship between $x_{\mathrm{sub}}^{i}$ and the substrate force $F_{\mathrm{sub}}^{i}$ as (figure 1*c*)

2.6 $$(K_{e}+K_{a})\gamma {\dot{x}}_{\mathrm{sub}}^{i}+K_{a}K_{e}x_{\mathrm{sub}}^{i}=K_{a}F_{\mathrm{sub}}^{i}+\gamma {\dot{F}}_{\mathrm{sub}}^{i},$$

where *K*_*e*_ is the elastic stiffness at *t* → ∞, *γ* is the substrate viscosity, and *K*_*a*_ is the additional stiffness that governs the substrate relaxation with a timescale *τ*_*r*_ ≡ *γ*/*K*_*a*_. A stress relaxation test from a constant strain yields the instantaneous and long-term stiffness of the substrate as *K*_*t*→0_ = *K*_*e*_ + *K*_*a*_ and *K*_*t*→∞_ = *K*_*e*_, respectively. The instantaneous stiffness *K*_*t*→0_ characterizes the initial elastic response of the substrate when the viscous deformation and stress relaxation have not yet taken place in the limit *t* → 0. Thus, cells with a very short focal adhesion lifetime (*τ*_*l*_ < *τ*_*r*_) can only sense the instantaneous stiffness. By contrast, the long-term stiffness *K*_*t*→∞_ refers to the residual substrate stiffness after the viscous stresses have relaxed.

#### 2.1.3. Cytoskeletal stiffening

The passively deforming cell cytoskeleton, which consists of microtubules and intermediate filaments, is represented by multiple springs in our model (figure 1*d*). Here we will assume that these cytoskeletal ‘springs’ undergo strain-stiffening during large spreading events and thus exhibit nonlinear elasticity. This assumption is backed by the experiments performed, e.g. on NIH-3T3 fibroblasts, that reveal a strong correlation between the cell rigidity and cell area during large spreading events [8]. Assuming this behaviour is mechanically driven and thus must be generic across animal cells, we propose a phenomenological equation for the cytoskeletal stiffness *K*_cs_ in terms of the cell area *A*: denoting the position vector of the cell nucleus by **x**_nuc_ and defining the length of a cytoskeletal spring as *r*^*i*^ ≡ |(**x**_nuc_ − **x**^*i*^)|, the cytoskeletal restoring force in our model is governed by the differential equation

2.7 $$\frac{dF_{\mathrm{cs}}^{i}}{dr^{i}}=K_{\mathrm{cs}}(A)=K_{\mathrm{cs}}^{b}+\Delta K_{\mathrm{cs}}\;e^{\beta A},$$

where $K_{\mathrm{cs}}^{b}$ denotes the baseline stiffness. The term Δ*K*_cs_ e^*βA*^ is introduced to describe the exponential hyperelastic behaviour [52,53], with the values of Δ*K*_cs_ and *β* obtained through linear regression of the experimental data in [8] (electronic supplementary material, §S3 and figure S6A).

#### 2.1.4. Local mechanical equilibrium

We enforce local force balance at each vertex to determine the radial protrusion force $F_{\;p}^{i}$ and the polar spreading speed $V_{s,\tau }^{i}$. The cell membrane possesses a notable mechanical rigidity that enables it to endure a variety of stresses, which is vital for maintaining the integrity of cell structures [41,54–56]. We model it as a closed loop of Hookean springs with stiffness *K*_*m*_ between neighbouring vertices. The tensile forces acting on the *i*th vertex can be expressed as $F_{m,\pm }^{i}=K_{m}(l_{\pm }^{i}-l_{0})$, where $l_{+}^{i}$ and $l_{-}^{i}$ are the distance vectors between vertex *i* and its two neighbours, and **l**_0_ is the initial distance between two adjacent vertices. The drag force on vertex *i* can be expressed as $F_{\eta }^{i}=\eta_{m}^{i}l^{i}V_{s}^{i}$, where $l^{i}\equiv (|l_{+}^{i}|+|l_{-}^{i}|)/2$ defines the average membrane length about the *i*th vertex. The drag force can originate, e.g. from the hydraulic resistance of the extracellular medium and the viscous response of the cortex beneath the cell membrane. Thus, the value of the drag coefficient $\eta_{m}^{i}$ in our model is within the range of the approximate hydraulic resistance coefficient and the range of the viscous coefficient used in other two-dimensional models (electronic supplementary material, §S4, figure S7) [54,56,57]. In addition to the membrane forces, each vertex experiences a protrusion force $F_{\;p}^{i}$ and a cytoskeletal radial force $F_{\mathrm{cs}}^{i}$. Thus, the net force balance at each vertex in the radial direction ${\hat{\;n}}^{i}$ and the polar direction ${\hat{\tau }}^{i}$ is given by

2.8*a* $$F_{\;p}^{i}-F_{\mathrm{cs}}^{i}+(F_{m,+}^{i}+F_{m,-}^{i})\cdot {\hat{n}}^{i}-\eta_{m}l^{i}V_{s,n}^{i}=0$$

and

2.8*b* $$(F_{m,+}^{i}+F_{m,-}^{i})\cdot {\hat{\tau }}^{i}-\eta_{m}l^{i}V_{s,\tau }^{i}=0.$$

Equations (2.8*a*) and (2.8*b*) yield $F_{\;p}^{i}$ and $V_{s,\tau }^{i}$, respectively. Altogether, equations (2.1)–(2.8b) fully determine the vertex spreading velocities $V_{s}^{i}$ when the nucleus displacement **x**_nuc_ and the dynamical GTPase concentrations at each vertex are computed at the cellular scale.

### 2.2. Equations of motion at cellular scale

Next, we explain the global mechanical equilibrium that governs the nucleus motion and the intracellular Rho-GTPase dynamics, which directly influence the aforementioned subcellular processes.

#### 2.2.1. Viscous drag on deformed nucleus

Our model quantifies cell translocation by the net translation of the cell nucleus, which balances the forces between the cytoskeletal microtubules and intermediate filament bundles. At the cell periphery, these cytoskeletal complexes are linked to the F-actin at the FA sites, transmitting the net traction force from the ECM ($F_{\mathrm{sub}}^{i}$) and the protrusion force from the cell boundary ($F_{\;p}^{i}$) to the cell nucleus (figure 1). This tight linkage to the rest of the cell can deform the nucleus when the cell flattens under large spreading on stiff substrates [29]. To balance these peripheral and cytoskeletal forces, the nucleus undergoes viscous drag within the cytoplasm while it deforms under cell flattening. The viscous drag force on a particle is commonly described by the product of a drag coefficient, a characteristic particle size and the velocity of the particle relative to the surrounding medium. Note that the cell peripheral dynamics cannot induce a net flow into the cell interior since the viscoelastic relaxation timescale of the cytoplasm *τ*_cp_ ∼ 0.1−1 s is much smaller than the cell migration timescale *T* ≡ *L*/*V*_0_ ≈ 260*s* (*L* = 2*πr*_0_: size of a circular cell, *V*_0_: retrograde flow speed; electronic supplementary material, table S1) [58]. As a result, flow around the nucleus should predominantly be induced by the dynamics of the nucleus and other organelles, as well as the cytoskeleton. In the absence of a detailed characterization of the organelles and the cytoskeleton, we assume that the drag force is only proportional to the nucleus speed ${\dot{x}}_{\mathrm{nuc}}$ [28,41,54]

2.9 $$F_{\mathrm{nuc}}^{\eta }=-f_{\mathrm{shape}}L_{\mathrm{nuc}}\eta_{\mathrm{cp}}{\dot{x}}_{\mathrm{nuc}},$$

where *η*_cp_ represents the viscosity of the cytoplasm and $L_{\mathrm{nuc}}$ is the characteristic particle size. For simplicity, we take $L_{\mathrm{nuc}}\equiv 6\pi r_{\mathrm{nuc}}^{0}$ corresponding to Stokes’ flow as a first-order approximation to the cytoplasmic domain within finite cell height ($r_{\mathrm{nuc}}^{0}$: the initial radius of the nucleus). The shape factor *f*_shape_ quantifies the deviation of the nucleus from a perfect sphere with *f*_shape_ = 1. The Corey shape function can be used to determine *f*_shape_ based on the nucleus’s three principal lengths (figure 1*e*) [59,60]

2.10 $$f_{\mathrm{shape}}={\left(\frac{l_{\max}l_{\mathrm{med}}}{l_{\min}^{2}}\right)}^{\alpha },$$

where the exponent *α* = 0.09 was obtained by fitting the experimental drag coefficient of non-spherical particles under Stokes flow [60]. For the deformed nucleus, the aspect ratio defined by the longest and the shortest dimensions can be related to the cell spreading area by *δ* ≡ *l*_max_/*l*_min_ = *χA* + 1, where the exponent *χ* = 0.0024 is obtained by fitting the experimental cell shape data in Li *et al*. [61] (electronic supplementary material, figure S6B). The intermediate dimension *l*_med_ corresponds to the length of the minor axis of the nucleus on the *x*–*y* plane, and it was found to have a constant ratio to the length of the major axis in experiments (*l*_med_ = 0.8*l*_max_) [29]. Consequently, the nucleus shape factor can be simply related to the cell spreading area *A* by

2.11 $$f_{\mathrm{shape}}=0.98{\left(\chi A+1\right)}^{2\alpha }.$$

Experiments have demonstrated that the cytoplasm viscosity, like the strain stiffening, is strongly correlated with the cell spreading area [31,32]. These experiments also indicate that the cell viscosity and cell stiffness exhibit the same trend with increasing substrate stiffness. Therefore, we assume an area-dependent cytoplasm viscosity (similar to that in equation (2.7)) as

2.12 $$\eta_{\mathrm{cp}}(A)=\eta_{\mathrm{cp}}^{0}+\Delta \eta_{\mathrm{cp}}\;e^{\beta A},$$

where $\eta_{\mathrm{cp}}^{0}$ is the cytoplasm viscosity on soft substrates [62] and Δ*η*_cp_ controls the rate of viscosity increase with the cell spreading area *A*. The fitting parameter Δ*η*_cp_ ensures that the maximum cytoplasmic viscosity is approximately twice its value on soft substrates as indicated by experiments [31]. The values of the Δ*η*_cp_ may vary depending on the cell type and physiological conditions, which could explain the quantitative differences in non-monotonic stiffness–speed relationships among different cell types [5,6,29].

At the frame of the nucleus, the condition for the mechanical equilibrium between equation (2.9), the cytoskeletal and the subcellular forces

2.13 $$\sum_{i=1}^{N}(F_{\mathrm{sub}}^{i}+F_{\mathrm{cs}}^{i}-F_{\;p}^{i}){\hat{n}}^{i}-F_{\mathrm{nuc}}^{\eta }=0$$

yields the nucleus migration velocity, ${\dot{x}}_{\mathrm{nuc}}$, equivalent to the cell velocity in our model.

#### 2.2.2. Reaction–diffusion dynamics of GTPase concentrations

As with our previous work [28], here we adopt the biochemical reaction–diffusion equations introduced in [63,64] to describe the dynamics of active and inactive GTPases. Since the active forms of the GTPases are predominantly associated with the cell membrane, which also serves as a major site for the conversion between active and inactive forms, we track the volume fractions of the signalling proteins in three forms [65,66]: the active membrane-bound form $\left(G_{a}^{i}(t)\equiv \{R_{a}^{i}(t),\rho_{a}^{i}(t)\}\right)$, the inactive membrane-bound form $\left(G_{\mathrm{in}}^{i}(t)\equiv \{R_{\mathrm{in}}^{i}(t),\rho_{\mathrm{in}}^{i}(t)\}\right)$, and the inactive cytosolic form (*G*_cp_(*t*) ≡ {*R*_cp_(*t*), *ρ*_cp_(*t*)}). Given the rapid diffusion of inactive proteins in the cytosol, we assume *G*_cp_(*t*) remains uniformly distributed in the cytosol at all times. Both active and inactive membrane-bound forms diffuse with a diffusion constant *D* across the vertices. The corresponding diffusive fluxes are given by Fick’s Law in a finite difference formulation as

2.14 $$J_{y}^{i}\equiv -D\left(\frac{G_{y}^{i+1}/l^{i+1}-G_{y}^{i}/l^{i}}{\left|l_{+}^{i}\right|}\right).$$

Equation (2.14) takes into account the effect of the deformed cell shape on the diffusive flux by updating the vertex coordinates at each time step. Different forms of the proteins on a vertex are interconvertible with the inactive-to-active rates $A_{G}^{i}$, active-to-inactive rates $I_{G}^{i}$, and inactive-to-cytosolic association and disassociation rates $M_{G}^{+}$, $M_{G}^{-}$ (figure 1*d*). Altogether, the reaction–diffusion kinetics is governed by

2.15 $$\begin{array}{rl} & {\dot{G}}_{a}^{i}=A_{G}^{i}G_{\mathrm{in}}^{i}-I_{G}^{i}G_{a}^{i}+(J_{a}^{i-1}-J_{a}^{i}),\\ & {\dot{G}}_{\mathrm{in}}^{i}=-A_{G}^{i}G_{\mathrm{in}}^{i}+I_{G}^{i}G_{a}^{i}+(J_{\mathrm{in}}^{i-1}-J_{\mathrm{in}}^{i})+\frac{M_{G}^{+}G_{\mathrm{cp}}}{N}-M_{G}^{-}G_{\mathrm{in}}^{i}\\ \mathrm{and}\; & {\dot{G}}_{\mathrm{cp}}=-M_{G}^{+}G_{\mathrm{cp}}+\sum_{i=1}^{N}M_{G}^{-}G_{\mathrm{in}}^{i}. \end{array}\}$$

The total amounts of Rac1 and RhoA are constant due to the conservation law $\sum_{i=1}^{N}{\dot{G}}_{a}^{i}+\sum_{i=1}^{N}{\dot{G}}_{\mathrm{in}}^{i}+{\dot{G}}_{\mathrm{cp}}=0$. The active Rac1 and RhoA GTPase volume fractions $R_{a}^{i},\rho_{a}^{i}$ regulate the cell migration by controlling the actin polymerization speed $V_{\;p}^{i}$ (equation (2.1)), the number of myosin motors $N_{m}^{i}$ (equation (2.2)) and the clutch number $N_{c}^{i}$ at each FA site (equation (2.5)). Different from Merchant *et al*. [63] and Zmurchok & Holmes [64], the rate terms $A_{G}^{i}$ in our formulation account for the reverse coupling from the cell deformation to the signalling pathways in addition to the mutual inhibition of the Rac1 and RhoA (appendix A.1). Since the filopodial protrusions of a mesenchymal cell grow and shrink at timescales comparable to the migration times, this chemo-mechanical feedback prevents the Rac1 and RhoA dynamics from reaching a steady bistable polarized state. Instead, when the transient chemical polarity ceases, our simulation algorithm reinstates the polarization stochastically to sustain the random migration patterns in many mesenchymal phenotypes [26,27].

### 2.3. Simulation procedure

We represent the initial vertex configuration of the cell as a regular 16-sided polygon based on the mesh convergence study in [28], which suggests that using *N* = 16–24 ensures decent numerical accuracy. All initial forces, velocities, displacements and substrate deformations are set to zero. Since chemical signalling drives cell polarization followed by directional locomotion on uniform substrates [28], a non-uniform initial distribution of the active Rac1 protein volume fraction $R_{a}^{i}$ is enforced with a higher value at the cell front. Likewise, a polarized initial distribution of the active RhoA protein volume fractions $\rho_{a}^{i}$ is taken with accumulation at the cell rear (manual polar symmetry breaking). In contrast, the initial conditions for $R_{\mathrm{in}}^{i}$, $\rho_{\mathrm{in}}^{i}$ are uniform at time *t* = 0, and the cytoplasmic form *R*_cp_, *ρ*_cp_ can be determined from the total volume fraction conservation (electronic supplementary material, figure S9). For directed migration on non-uniform substrates, uniform initial signalling distributions must be assumed since the polarity will be set by the gradients of the ECM stiffness or viscosity. Starting from these initial conditions, the vertex spreading and the nucleus velocities are calculated by solving equations (2.1)–(2.13), and the Rho-GTPase volume fractions are updated by solving equations (2.14), (2.15), (A 1) and (A 2) by following the algorithm in electronic supplementary material, figure S4. When the cytoplasmic inactive Rac1 volume fraction *R*_cp_ reaches a steady state, we reinitialize $R_{a}^{i}$ and $\rho_{a}^{i}$ stochastically to mimic the random nature of the protrusion formations in the mesenchymal cells. To incorporate the adhesion reinforcement (equation (2.3)), we fix *ζ* = 0.5 pN^−1^ since it leads to a good agreement between the simulated spreading areas and the experimental data in Solon *et al*. [8] (electronic supplementary material, figure S10). The parameters Δ*K*_cs_ = 2.8 pN μm^−1^ and *β* = 0.00185 (equation (2.7)) and *χ* = 2.4 × 10^−3^ (equation (2.11)) provide the best fit to the experimental data from Solon *et al*. [8] and are kept constant in all simulations (electronic supplementary material, figure S6). All fixed simulation parameters are listed in electronic supplementary material, table S1.

We present the simulation results in dimensionless form by introducing a position scale *L* ≡ 2*πr*_0_ = 10*π* μm, retrograde flow speed scale *V*_0_ = 120 nm s^−1^, migration timescale *T* ≡ *L*/*V*_0_ ≈ 260 s and force scale $F_{0}\equiv N_{m}^{0}f_{m}=200\;\mathrm{pN}$. For each simulation, we run *M* > 10^6^ time steps with a time step of approximately Δ*t* = 1.5 × 10^−3^ s (Δ*t* ≈ 5.7 × 10^−6^ in dimensionless units), corresponding to the migration dynamics over at least an hour in real units. The spreading area *A* is calculated as the average cell area over the last 10^6^ time steps in a simulation. We define the migration speed *V* as the nucleus trajectory length *X* ≡ |**x**_nuc_(*M*Δ*t*) − **x**_nuc_(0)| divided by the total time *t*_total_ ≡ *M*Δ*t*, i.e. *V* ≡ *X*/*t*_total_. The ranges of the ECM material parameters used in the simulations are listed in table 1. For each set of the ECM parameters, we run *n* simulations to compute the sample mean spreading area, $\bar{A}$, and the mean migration speed, $\bar{V}$. The effects of the ECM viscoelasticity on the spreading area, migration speed and migration direction are validated by the experimental data taken from [6,16,27].

**Table 1.** Residual substrate stiffness *K*_*e*_, additional stiffness *K*_*a*_ and viscosity *γ* ranges in simulations.

parameter	real units	dimensionless units
*K*_*e*_	0.1–100 pN nm^−1^ [17,23,46]	5*π*–5000*π*
*K*_*a*_	0.1–20 pN nm^−1^	20*π*–4000*π*
*γ*	0.01–100.0 pN s nm^−1^ [23]	0.06–60

## 3. Results

### 3.1. Simulations reproduce experimental cell spreading and migration speed dependence on substrate stiffness

**Figure 2.**
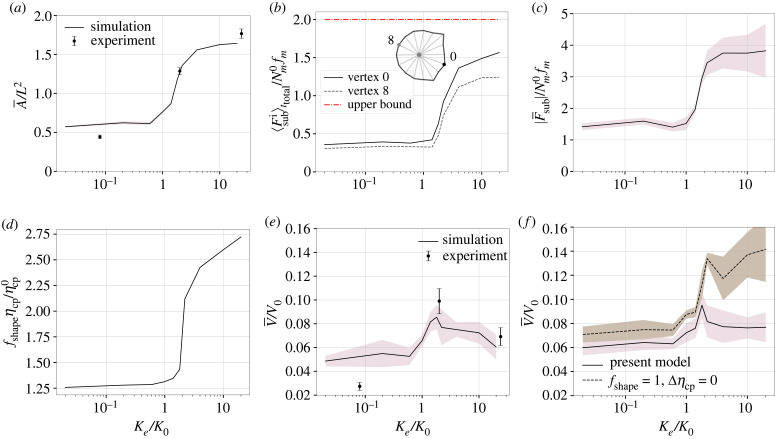
Cell spreading area and migration speed on elastic substrates. (*a*) The dimensionless mean cell area $\bar{A}/L^{2}$ (full line) as a function of the dimensionless substrate elastic stiffness *K*_*e*_/*K*_0_. For comparison, the dots and error bars are the experimental data from [6].^1^ (*b*) The average dimensionless substrate traction force versus *K*_*e*_/*K*_0_ at vertex 0 (cell front) and vertex 8 (cell rear). The red dash-dotted line gives the upper bound of the substrate traction force on very stiff substrates. The calculation of the averaged substrate traction forces ${\left\langle F_{\mathrm{sub}}^{i}\right\rangle }_{t_{\mathrm{total}}}$ from the full focal adhesion cycles is illustrated in electronic supplementary material, figure S11. (*c*) The dimensionless mean and standard deviations of the net traction force versus *K*_*e*_/*K*_0_. The calculation of the time-averaged net traction force is illustrated in electronic supplementary material, figure S12. (*d*) Cell-spreading induced nucleus viscous drag increase $f_{\mathrm{shape}}\eta_{\mathrm{cp}}/\eta_{\mathrm{cp}}^{0}$ versus *K*_*e*_/*K*_0_, which is calculated by using equations (2.9), (2.11), (2.12) for the mean spreading area values in *a*. (*e*) The simulated dimensionless mean migration speed $\bar{V}/V_{0}$ versus *K*_*e*_/*K*_0_ (full curve) and the experimental data for the U373-MG human glioma cells (dots and error bars) [6]. (*f*) The influence of the shape factor *f*_shape_ of the deformed nucleus and the area-dependent cytoplasm viscosity *η*_cp_ on the migration speed (equations (2.11), (2.12)). The mean values or the standard deviations are calculated over *n* = 10 simulations at each data point in *a*, *c*–*f*.

With increasing stiffness *K*_*e*_ on an elastic substrate (*γ* = 0, *K*_*a*_ = 0), the cell spreading area increases monotonically, in agreement with the experiments on the adhesion-reinforced U373-MG human glioma cell spreading on polyacrylamide hydrogels (figure 2*a*) [6]. This can be understood by inspecting the FA dynamics at the subcellular scale: according to equations (2.2), (2.5), the cell spreading speed at a vertex $V_{s,n}^{i}$ is set by the competition between the polymerization speed $V_{\;p}^{i}$ and the actin retrograde flow speed $V_{r}^{i}$, which is counteracted by the stiffness-dependent focal traction force (appendix A.2)

3.1 $$F_{\mathrm{sub}}=N_{m}^{0}f_{m}(1-e^{-t/\tau_{l}}),\;\tau_{l}\equiv \frac{N_{m}^{0}f_{m}}{V_{0}K_{e}}.$$

Equation (3.1) is derived for an isolated motor-clutch system where the engaged clutch fraction is assumed to be saturated on a compliant substrate (*P*^*i*^ ≈ 1; $K_{e}\ll N_{c}^{0}K_{c}$). For sufficiently low *K*_*e*_ where the adhesion reinforcement mechanism is not triggered (see equation (2.3)), the traction force starts building up at an FA site after the molecular clutches form at a timescale $\tau_{\mathrm{on}}^{0}\equiv 1/k_{\mathrm{on}}^{0}$, and develops with a characteristic time *τ*_*l*_ (equation (3.1)). The comparison between the two timescales $\left(\tau_{\mathrm{on}}^{0}=\tau_{l}\right)$ defines a threshold stiffness $K_{0}\equiv N_{m}^{0}f_{m}k_{\mathrm{on}}^{0}/V_{0}\approx 5\;{\mathrm{pN}\;\mathrm{nm}}^{-1}$ (table 2). On soft substrates (*K*_*e*_ ≤ *K*_0_), since the force build-up time *τ*_*l*_ is much longer than the clutch formation time *τ*_on_, the adhesion force mostly remains low, i.e. $F_{\mathrm{sub}}^{i}<N_{m}^{0}f_{m}$ (equation (3.1); figure 2*b*). For a stiff substrate with *K*_*e*_ > *K*_0_, the decrease in the force build-up time *τ*_*l*_ must cause a steep increase in the average force per bond (${\left\langle f_{a}\right\rangle }_{\tau_{l}}>f_{\mathrm{cr}}$), triggering the adhesion reinforcement mechanism with an augmented binding rate *k*_on_ (equation (2.3), electronic supplementary material, figure S3). The reinforced binding rate can even saturate the bounded clutches on a very stiff substrate, i.e. *P*^*i*^ → 1 (electronic supplementary material, figure S3F). Moreover, a higher ${\left\langle f_{a}\right\rangle }_{\tau_{l}}$ on stiff substrates leads to higher disassociation rates *k*_off_, which must balance the binding rate in equation (2.4) to reach a steady bound clutch fraction *P*^*i*^ ∼ 1. This condition yields an estimate for the instantaneous force per bond as $f_{c}^{i}\propto \zeta ({\left\langle f_{a}\right\rangle }_{\tau_{l}}-f_{\mathrm{cr}})f_{r0}$ (appendix A.3). This allows us to estimate an upper bound of the substrate traction force on a stiff substrate as $F_{\mathrm{sub}}^{i}=N_{c}^{i}f_{c}^{i}\approx 8\zeta N_{c}^{i}>N_{m}^{0}f_{m}$, in agreement with the simulations (figure 2*b*). This elevated traction at *K*_*e*_ > *K*_0_ across the cell is the main driving factor behind enhanced cell spreading. Furthermore, the Rho GTPase polarization leads to a higher traction force at the cell front than at the rear since the motor-clutch number $N_{c}^{i}$ is controlled by the Rac1 concentration (figure 2*b*). Owing to the chemically induced polarization, the cell acquires a larger net traction force on stiff substrates than on soft ones (figure 2*c*).

**Table 2.** Characteristic scales for spreading and migration dynamics. For a viscoelastic substrate, *K*_*a*_ is the additional stiffness, and *γ* is the viscosity in the SLS model. Additionally, $N_{m}^{0}$ is the reference myosin motor number, *V*_0_ is the unloaded myosin motor speed, *f*_*m*_ is the force that stalls the activity of one myosin motor, and $k_{\mathrm{on}}^{0}$ denotes the reference clutch binding rate.

quantity	clutch binding timescale^a^	clutch lifetime^a^	threshold stiffness	substrate relaxation timescale	optimal viscosity
definition	$\tau_{\mathrm{on}}^{0}\equiv 1/k_{\mathrm{on}}^{0}$	$\tau_{l}\equiv N_{m}^{0}f_{m}/V_{0}K_{e}$	$K_{0}\equiv N_{m}^{0}f_{m}k_{\mathrm{on}}^{0}/V_{0}$	*τ*_*r*_ ≡ *γ*/*K*_*a*_	$\gamma_{0}\equiv K_{a}/k_{\mathrm{on}}^{0}$
value	1/3 s	1/3–50/3 s	≈5.0 pN nm^−1^	10^−2^–10^2^ s	≤5/3 pN · s nm^−1^

^a^The scales are valid for substrates with a stiffness lower than *K*_0_.

The cell motility is governed by the competition between the net traction force and the viscous drag of the nucleus since the remaining forces are negligible in equation (2.13) (electronic supplementary material, figure S13). Because large cell spreading on stiff substrates can deform the nucleus (figure 2*d*) and alter the cytoplasmic viscosity (equation (2.12)), the viscous drag force on the nucleus must increase, resisting the net traction force. Consequently, the migration efficiency decreases beyond the threshold stiffness *K*_0_, creating a non-monotonic stiffness–speed profile, which also quantitatively reproduces the U373-MG human glioma cell speed on polyacrylamide hydrogels (figure 2*e*) [6]. In our simulations, ignoring the nucleus shape change and assuming constant cytoplasmic viscosity recovers a monotonic relationship between the migration speed and substrate stiffness, which validates their role in the non-monotonic speed–stiffness relation (figure 2*f*). Furthermore, we have performed parametric studies to explore the relative impact of the nucleus shape change and the cytoplasmic viscosity increase on the non-monotonic speed–rigidity relationship (electronic supplementary material, figure S14). We found that while both factors influence the migration speed on soft substrates, the effect of cytoplasmic viscosity increase becomes more significant on stiff substrates, emphasizing its critical role in capturing the non-monotonic relationship. We also examined the potential impact of cytoplasmic flows on nucleus motion by modifying equation (2.9) to consider the centroid velocity. Electronic supplementary material, figure S8 demonstrates that this approximation does not substantially influence the efficiency of cell movement over long times. Overall, because glioma cells can migrate with or without adhesion reinforcement [16,69], our current results complement our previous work in [28] by explaining how a non-monotonic speed–stiffness relation emerges while the traction forces remain monotonic.

### 3.2. Substrate viscosity affects cell spreading area and migration speed

We next discuss the effect of substrate viscosity on the cell area and migration speed on a substrate that is soft in the long time limit, *K*_*e*_ = 0.1 pN nm^−1^. When *K*_*e*_ + *K*_*a*_ < *K*_0_ (soft regime), the adhesion reinforcement effect can be neglected even on viscous substrates. However, when *K*_*e*_ + *K*_*a*_ ≥ *K*_0_ (stiff regime), a large viscosity may trigger the adhesion reinforcement regime and alter the spreading behaviour. We discuss both regimes below.

**Figure 3.**
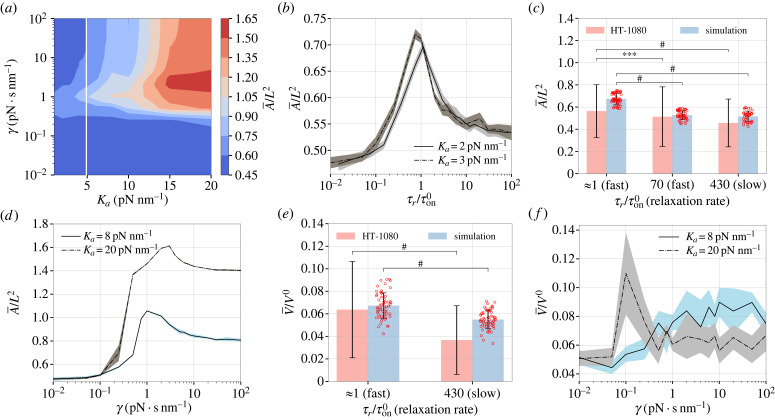
Effect of substrate viscosity on spreading area and migration speed. (*a*) Contour plot of the mean dimensionless cell area $\bar{A}/L^{2}$ on viscoelastic substrates as a function of the additional stiffness *K*_*a*_ and substrate viscosity *γ* (*K*_*e*_ = 0.1 pN nm^−1^). The white line denotes the threshold stiffness *K*_0_. (*b*) $\bar{A}/L^{2}$ and its standard deviation (shaded area) versus the ratio of the material relaxation timescale *τ*_*r*_ ≡ *γ*/*K*_*a*_ to the clutch binding timescale $\tau_{\mathrm{on}}^{0}\equiv 1/k_{\mathrm{on}}^{0}$ on soft substrates. (*c*) For a fixed instantaneous stiffness *K*_*e*_ + *K*_*a*_ = 2.6 pN nm^−1^, the simulated spreading area $\bar{A}$ and its standard deviation (*n* > 50) and experimental data for HT-1080 cells (data from [27]). By fitting the stress-time data in the stress relaxation test, the viscosities of the fast, intermediate (med) and slow relaxing substrates are found as *γ*_fast_ = 1 pN s nm^−1^, *γ*_med_ = 60 pN s nm^−1^, *γ*_slow_ = 360 pN s nm^−1^ (electronic supplementary material, figure S16). (*d*) The dimensionless spreading area $\bar{A}/L^{2}$ and its standard deviation as a function of *γ* for different *K*_*a*_ > *K*_0_. Since the clutch binding timescale *τ*_on_ depends on the adhesion reinforcement on stiff substrates, $\bar{A}/L^{2}$ is plotted against the substrate viscosity *γ*. (*e*) The mean migration speed $\bar{V}$ and its standard deviation in the simulations (*n* > 50) versus experiments of HT-1080 cells (data from [27]) on fast-relaxing and slow-relaxing soft substrates, for which the material parameters are extracted from fitting the stress-time data (electronic supplementary material, figure S16). (*f*) The dimensionless mean migration speed $\bar{V}/V_{0}$ and its standard deviation as a function of *γ* for two additional stiffness values *K*_*a*_ = 8 pN nm^−1^ and *K*_*a*_ = 20 pN nm^−1^. The contour plot in (*a*) contains 180 data points. The mean and standard deviations are calculated with *n* ≥ 5 simulations at each point in *b*, *d*, *f*. In *c*, *e*, statistical analyses were conducted using the *t*-test to compare simulation results with experimental data. Levels of statistical significance (*p*) are indicated as follows: ${\;}^{\#}p<0.0001$ and ***p < 0.001.

On substrates with a low instantaneous stiffness (*K*_*e*_ + *K*_*a*_ < *K*_0_), the cell spreading area varies in a non-monotonic manner with increasing viscosity (figure 3*a*,*b*). This can be explained by examining the FA dynamics at the subcellular scale, similar to the cell spreading on elastic substrates. At an FA site without adhesion reinforcement, time averaging the traction force (see electronic supplementary material, figure S15A,B) over a clutch lifetime *τ*_*l*_ leads to (appendix A.2)

$${\left\langle F_{\mathrm{sub}}^{i}\right\rangle }_{\tau_{l}}\approx \left\{ \begin{array}{cc} N_{m}^{0}f_{m}\exp\left[\frac{-\tau_{l}}{\tau_{l}+\tau_{r}(1+(K_{a}/K_{e}))}\right],\;\text{if}\;\tau_{r}\leq \tau_{\mathrm{on}}^{0} & \left(3.2a\right)\\ N_{m}^{0}f_{m}\exp\left(-1\right),\;\text{if}\;\tau_{r}\geq \tau_{l}, & \left(3.2b\right) \end{array}\right.$$

where the timescales $\tau_{l},\tau_{\mathrm{on}}^{0},\tau_{r}$ are defined in table 2. Equation (3.2*a*) indicates that the average traction force at an FA site must increase with increasing viscosity when $\tau_{r}\leq \tau_{\mathrm{on}}^{0}$. At $\tau_{r}>\tau_{\mathrm{on}}^{0}$, since slower substrate relaxation leads to the FA lifetime reduction when (electronic supplementary material, figure S15C) the average traction force should exhibit a decreasing profile with a lower bound that is estimated in equation (3.2*b*). That way, equation (3.2) indicates the presence of a non-monotonic traction force–viscosity profile in an isolated motor-clutch system on soft substrates. Note that when $\tau_{\mathrm{on}}^{0}<\tau_{r}<\tau_{l}$, the average substrate force is not amenable to an analytical approximation. Still, given the biphasic profile suggested by equation (3.2), it is plausible to assume that the average substrate force reaches a maximum when $\tau_{r}\approx \tau_{\mathrm{on}}^{0}$, equivalently at an optimal viscosity $\gamma_{0}\equiv K_{a}\tau_{\mathrm{on}}^{0}$ (electronic supplementary material, figure S15C), thereby maximizing the cell spreading area. To further validate our model, we simulated spreading areas of human fibrosarcoma cells HT-1080 on viscoelastic substrates consisting of interpenetrating alginate networks (IPN) [27]. The experimental material parameters of the substrates with different relaxation rates were obtained by data fitting to the stress relaxation tests in [27] (electronic supplementary material, §S3 and figure S16). Clearly, our whole-cell model accurately replicates the experimentally observed spreading areas of the HT-1080 cells on soft substrates with different stress relaxation rates (figure 3*c*), indicating that cell spreading is suppressed with increasing viscosity when $\tau_{r}>\tau_{\mathrm{on}}^{0}$.

At a larger instantaneous substrate stiffness *K*_*e*_ + *K*_*a*_ ≥ *K*_0_, the cells only perceive the long-term stiffness *K*_*e*_ and maintain small spreading areas at a low substrate viscosity *γ* (figure 3*d*). For higher *γ*, the slowly relaxing substrate maintains the stiffness *K*_*e*_ + *K*_*a*_ ≥ *K*_0_ at later times, sustaining the adhesion reinforcement regime and thus promoting cell spreading. Moreover, because the adhesion reinforcement effect becomes more pronounced on stiffer substrates, a larger additional stiffness, such as *K*_*a*_ = 20 pN nm^−1^ further increases the cell spreading area at high *γ* (figure 3*d*).

As opposed to the spreading area that can be understood at the single FA level, the change in the cell migration speed as a function of *γ* must be examined by virtue of the net traction force $\sum_{i}F_{\mathrm{sub}}^{i}$ at the cellular scale. At an instantaneous substrate stiffness *K*_*a*_ + *K*_*e*_ < *K*_0_, where the adhesion reinforcement is not activated, the high viscosity of *γ* > *γ*_0_ can result in a decrease of the FA lifetime and thus a lower traction force (appendix A.2 and electronic supplementary material, figure S15D). Our simulations reveal that migration on fast relaxing substrates ($\tau_{r}∼\tau_{\mathrm{on}}^{0}$) is more efficient than on slow relaxing substrates ($\tau_{r}\gg \tau_{\mathrm{on}}^{0}$) (figure 3*e*). This is in quantitative agreement with the migration speeds of the MDA-MB-231 human breast cancer adenocarcinoma cells on fast and slow relaxing IPN [27]. A more comprehensive parametric study demonstrates the presence of a biphasic speed–viscosity relation (electronic supplementary material, figure S17A,B). The optimal viscosity corresponding to the maximum migration speed tends to be slightly higher than *γ*_0_ (for maximum cell spreading), primarily due to the prolonged FA lifetime at the front of the cell (appendix A.2). On substrates with high instantaneous stiffness (*K*_*a*_ + *K*_*e*_ ≥ *K*_0_), the net traction force increases with viscosity due to the adhesion reinforcement effect (electronic supplementary material, figure S17C). Since cells have limited spreading areas due to the weak engagement of the adhesion reinforcement regime at *K*_*a*_ = 8 pN nm^−1^ (figure 3*d*), the change in the nucleus drag force is marginal. Thus, we obtain a nearly monotonic increase in the migration speed with viscosity (figure 3*f*). However, with a very large additional stiffness of *K*_*a*_ ≫ *K*_0_, strong adhesion reinforcement on the viscous substrate results in very large spreading areas (figure 3*d*), which significantly increases the nucleus drag forces and in turn leads to a biphasic speed–viscosity profile (figure 3*f*).

### 3.3. Migration direction is set by stiffness or viscosity variation on non-uniform substrates

**Figure 4.**
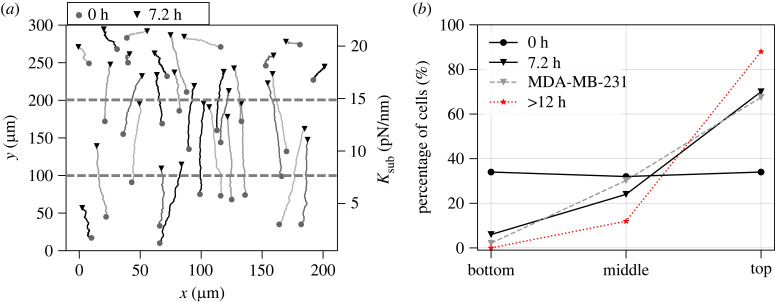
Simulations reproduce cell durotaxis on elastic gradient substrates. (*a*) Trajectories of 30 simulated cells over greater than 7.2 h in real units on an elastic substrate with a linear stiffness variation between *K*_*e*_ = 0.5–22 pN nm^−1^ in the *y*-direction. (*b*) The percentage of the simulated cells out of *n* = 83 simulations on the bottom, middle and top third of the gradient substrate as a function of time, i.e. at *t* = 0 h (initial condition), *t* = 7.2 h, *t* > 12 h, which are compared with the distributions of the MDA-MB-231 cells (grey dashed lines) that are seeded on polyacrylamide hydrogels with a stiffness gradient and observed for 72 h [16].

Having elucidated the effect of substrate stiffness and viscosity on cell area and migration speed, we investigate the sensitivity of cell migration to a stiffness gradient on elastic substrates or a viscosity gradient on viscoelastic substrates. To eliminate the effect of chemical polarity on migration direction in the simulations, we specify the initial conditions of a chemical apolar cell by assigning a random distribution of the active Rac1 concentration $R_{a}^{i}$ and setting the other membrane-bound Rho GTPase concentrations $\rho_{a}^{i}$, $R_{\mathrm{in}}^{i}$, $\rho_{\mathrm{in}}^{i}$ constant (electronic supplementary material, figure S18). Migrating cells exhibit limited migration speeds ($\bar{V}<50\;\mu {m\;h}^{-1}$), leading to a substantial amount of time required for simulating cell locomotion over long distances. Therefore, for the sake of computational efficiency, we adopt a 200 × 300 μm domain at about a 1 : 10 scale of the experimental platform (figure 4*a*). We compared our migration simulations of 83 individual cells on elastic gradient substrates with the experimental migration patterns of the MDA-MB-231 cells on polyacrylamide hydrogels with a stiffness gradient 0.5–22.0 kPa [16]. We record the migration trajectory of each simulated cell for 7.2 h, which is 1/10 of the experimental observation time (72 h). Owing to the difficulty in quantifying the substrate gradient in the experiments, we assume a constant gradient $\nabla K_{e}\approx 72$ pN nm^−2^ in the +*y*-direction in the simulations; non-dimensionalization by the initial cell diameter 2*r*_0_ and the threshold stiffness *K*_0_ yields the unitless gradient $\tilde{\nabla }\tilde{K_{e}}\approx 0.14<1$. The simulated cell trajectories indicate that all cells with random locations at *t* = 0 move up the stiffness gradient regardless of their initial positions, i.e. the cells undergo robust durotaxis (figure 4*a*). Also, the percentages of the simulated cells in three substrate portions demonstrate the strong migration trend towards the stiffest region (figure 4*b*), in good agreement with the gradient sensitivity of the MDA-MB-231 cells with talin- and vinculin-mediated FA formations [16]. We further confirm that almost all cells eventually migrate to the stiffest region after more than 12 h (figure 4*b*). The robust durotaxis behaviour is mainly due to the monotonic increase of the traction force as a function of the substrate stiffness at an FA site due to the adhesion reinforcement (figure 2*b*). In other words, the FA sites attached to the stiffer regions generate a higher traction force than those attached to the softer regions on a non-uniform substrate, driving the cell up the stiffness gradient.

**Figure 5.**
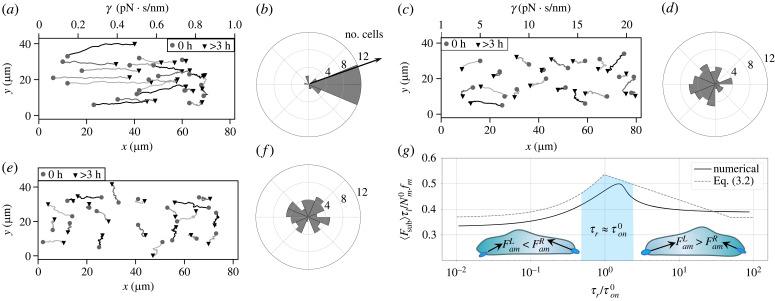
Directed migration is regulated by viscosity gradient on soft substrates. Representative trajectories of 20 cells and angular displacements of approximately 50 cells on a viscoelastic substrate (*K*_*a*_ = 0.1 pN nm^−1^, *K*_*e*_ = 2.9 pN nm^−1^) with (*a*), (*b*) a linearly varying viscosity *γ* = 0.0–1 pN s nm^−1^, (*c*), (*d*) *γ* = 1.0–20 pN s nm^−1^, and (*e*), (*f*) a uniform viscosity *γ* = 10 pN s nm^−1^. (*g*) The simulated versus analytical traction forces (averaged over *τ*_*l*_) as a function of the ratio of the material relaxation timescale *τ*_*r*_ ≡ *γ*/*K*_*a*_ to the clutch binding timescale $\tau_{\mathrm{on}}^{0}\equiv 1/k_{\mathrm{on}}^{0}$ (table 2). The traction forces at the two opposite ends of a cell along the direction of the motion are labelled by $F_{\mathrm{am}}^{R}$ or $F_{\mathrm{am}}^{L}$, respectively.

We next sought to understand the impact of substrate stress relaxation on the directed migration of cells on soft substrates. In our simulations, the two stiffness values in the SLS model are set to be *K*_*a*_ = 2.9 pN nm^−1^ and *K*_*e*_ = 0.1 pN nm^−1^. We assume a non-uniform viscosity *γ* < *γ*_0_ ≈ 1 pN s nm^−1^ with a constant gradient $\nabla \gamma =12.5\;\mathrm{pN}\;s\;{\text{μ}m}^{-2}$ in +*x*-direction, equivalent to $\tilde{\nabla }\tilde{\gamma }\approx 0.125$ in the unitless form (non-dimensionalized by *γ*_0_ and 2*r*_0_). For computational efficiency, we used a 80 × 40 μm domain, which still much larger than a migrating cell size in ±*x*-direction. The simulations demonstrate that cells migrate from the soft elastic region (*γ* → 0) to the viscoelastic region, which is also evidenced by the biased angular displacement towards the +*x*-direction (figure 5*a*,*b*). The same trend was observed in human mesenchymal stem cells that migrate towards regions with a larger relaxation modulus on a collagen-coated polyacrylamide gel. This behaviour is referred to as viscotaxis [39]. Once the substrate viscosity surpasses the threshold (*γ* > *γ*_0_), cells migrate against the viscosity gradient with the angular displacements biased toward faster relaxation regions, which can be referred to as ‘anti-viscotaxis’ (figure 5*c*,*d*). As a control, the random cell trajectories and unbiased angular displacements were observed on a uniform viscoelastic substrate (figure 5*e*,*f*). These findings suggest that a viscosity gradient draws the cells to a region with $\tau_{r}\approx \tau_{\mathrm{on}}^{0}$. The underlying mechanism for the dependence of the migration direction on the viscosity gradient can be explained by inspecting the biphasic viscosity–traction force relationship at an FA site. On soft substrates with a relaxation timescale $\tau_{r}<\tau_{\mathrm{on}}^{0}$, an increasing viscosity contributes to an enhanced substrate traction force that counteracts retrograde actin flow. This leads to a substrate-induced polarity along the gradient. By contrast, slower relaxing substrates ($\tau_{r}>\tau_{\mathrm{on}}^{0}$) lead to decreasing clutch lifetime (electronic supplementary material, figure S15C) and the reduced mean traction forces (equation (3.2)). As a result, the edges of the cell attached to the faster relaxing substrate regions gain more traction, navigating the cell against the viscosity gradient (figure 5*g*).

## 4. Discussion

Our multiscale theory involves a detailed description of the adhesion reinforcement regime at each FA site and includes the cytoskeleton strain-stiffening effect, which quantitatively reproduces the large cell spreading areas on stiff substrates. Importantly, by incorporating the viscous drag force increase due to the nucleus shape change and nonlinear cytoplasmic viscosity as a function of the spreading area, we have reproduced the non-monotonic dependence of the migration speed on substrate stiffness. Our findings indicate that the interplay among larger spreading areas on stiff substrates, variable cytoplasmic viscosity, and nucleus flattening can influence the drag forces in cell migration and give rise to the non-monotonic speed profile. Therefore, the mechanism underlying the non-monotonic speed–stiffness relation in this study is distinct from the biphasic speed-stiffness relation discussed in our previous work [28]. The biphasic speed profile in [28] is attributed to the biphasic dependence of the focal adhesion force on substrate stiffness in cells that lack an adhesion reinforcement. A limitation of our previous model was that it cannot reproduce the monotonic increase of spreading area with stiffness. By contrast, our current model effectively reconciles both the observed monotonic spreading area–stiffness relationship and the non-monotonic speed–stiffness relationship, as reported in many experiments [5–7]. Another important addition in our current study compared with our previous model is the inclusion of the cytoskeletal strain-stiffening effect. This effect plays a crucial role in accurately quantifying both cell spreading and migration efficiency of cells with adhesion reinforcement (see electronic supplementary material, figure S19). Ignoring this effect could lead to a significant overestimation of the spreading area on stiff substrates, leading to a migration behaviour that nearly comes to a halt.

Our simulations on viscoelastic substrates also reveal the influence of substrate viscosity on cell migration by tuning the engagement of the adhesion reinforcement regime. On a substrate with low instantaneous stiffness (*K*_*a*_ + *K*_*e*_ < *K*_0_) where the adhesion reinforcement regime is absent, we have analytically shown the presence of a biphasic traction force–viscosity relationship at an FA site, with the traction force maximized on fast-relaxing substrates ($\tau_{r}\approx \tau_{\mathrm{on}}^{0}$). The analytical relation helps us explain the simulation results, which quantitatively agree with the experiments. On substrates with a large instantaneous stiffness (*K*_*a*_ + *K*_*e*_ ≥ *K*_0_), the substrate viscosity can greatly enhance the cell spreading area due to the engagement of the adhesion reinforcement regime. Notably, although the net traction force also increases with viscosity, we observe a biphasic speed–viscosity profile with a very large additional stiffness of *K*_*a*_ ≫ *K*_0_. This is because high viscosity on a very stiff substrate triggers strong adhesion reinforcement, which induces a large cell spreading area, in turn raising the nucleus drag force that counteracts the net traction force.

Finally, we have investigated the influence of substrate viscoelasticity on directed migration. Our model successfully captures the robust durotaxis behaviour of the MDA-MB-231 cells with talin-/vinculin-mediated clutch reinforcement on elastic gradient substrates. These results complement our previous findings regarding the talin-low cells that operate without adhesion reinforcement and thus exhibit the co-occurrence of durotaxis and anti-durotaxis on gradient substrates. As a result, our investigations have successfully replicated the complete spectrum of directed migration behaviours displayed by MDA-MB-231 cells reported in [16]. This underscores the pivotal influence of the adhesion reinforcement regime in governing cell durotactic responses on gradient substrates. Furthermore, our simulated cells also exhibit both viscotaxis and anti-viscotaxis, with all cells migrating towards the fastest-relaxing substrate region where the traction force at an FA site is maximum when $\tau_{r}\approx \tau_{\mathrm{on}}^{0}$. This reveals the critical role of stress relaxation in modulating the migration direction.

Our framework has inherent physical and mathematical limitations in that it is built by coupling cellular processes that are not yet fully understood. For instance, the model only takes into account the active regimes of the actin cytoskeleton at the sub-cellular level using the motor-clutch model. A rigorous modelling of the actin cytoskeleton could involve adopting the active gel theory, which characterizes dynamic structures formed by the interactions of actin filaments and associated proteins [70–72]. Moreover, the interplay between the cytoplasmic flow and the actin cytoskeleton plays a pivotal role in cellular function and behaviour [73]. While we show the minimal effect of cytoplasmic flows on the cell migration speed by approximating the net cytoplasm velocity using the centroid velocity (electronic supplementary material, figure S8), it is important to clarify that this approximation does not physically represent the cytoplasmic flow that is governed by the dynamics of cytoskeletal elements and the resulting fluid–structure interactions. Incorporating fluid mechanics into the active gel theory of the cytoskeleton can also enrich our comprehension of the dynamic nature of cells and enable us to understand certain unresolved phenomena, such as the transition between amoeboid and mesenchymal migration [74,75].

These limitations notwithstanding, our theory provides a valuable tool to understand how the intricate interplay between cellular signalling and mechanics along with cell–ECM interactions regulates migration on viscoelastic substrates. Hence, the theory paves the way for the development of novel experimental platforms for the manipulation of cell migration patterns.

## Data Availability

The Python source codes of the simulations can be accessed from the Dryad digital repository: https://doi.org/10.5061/dryad.h9w0vt4qr. Supplementary material is available online [76].

## Footnotes

^1^
We converted the experimental substrate modulus *E*_sub_ (kPa) to the stiffness *K*_sub_ (pN nm^−1^) by using the relation *K*_sub_ ≈ 2 *E*_sub_ *R*_FA_, where 2 *R*_FA_ ≈ 1.0 μm approximates the diameter of the nearly circular FA region [67,68].

## Appendix A

**A.1. Rate coefficients in Rho GTPase reaction–diffusion model**

Definitions of rates $A_{G}^{i}$ in equation (2.15) accommodate autoactivation and antagonistic effects as well as the feedback from the mechanical deformations of the cell membrane. The RhoA activation rate $A_{\rho }^{i}$ at the *i*th vertex is composed of three terms,

A 1 $$A_{\rho }^{i}=\frac{\alpha_{\rho }{\rho_{a}^{i}}^{3}}{\left({\rho_{0}}^{3}+{\rho_{a}^{i}}^{3}\right)}+\frac{\beta_{\rho }{R_{0}}^{3}}{\left({R_{0}}^{3}+{R_{a}^{i}}^{3}\right)}+e^{-H(-\Delta \theta^{i}-c_{0}\theta_{0})\frac{\Delta \theta^{i}}{\theta_{0}}}\kappa_{b}^{+},$$

where H() denotes the Heaviside step function. The reference levels of the active Rac1 and RhoA concentrations on a vertex are, respectively, labelled by *R*_0_ and *ρ*_0_, which are determined by the initial average fractions of $R_{a}^{i}$ and $\rho_{a}^{i}$ across the cell periphery. The first term takes into account positive feedback from the active RhoA itself. The magnitude of the autoactivation effect is represented by *α*_*ρ*_ [77,78]. The second term describes the mutual inhibition effect between Rac1 and RhoA with a rate *β*_*ρ*_ [77,78]. The last term couples the active level of RhoA to the mechanical deformations of the cell at the *i*th vertex, which is motivated by previous studies demonstrating the significance of a two-way coupling between the Rho GTPases and mechanical forces through one-dimensional migration modelling [14,78]. The initial angle at a cell vertex is given by *θ*_0_ = 7*π*/8, for a regular 16-sided polygon, and Δ*θ*^*i*^ ≡ *θ*^*i*^ − *θ*_0_ denotes the change in angle at the vertex (figure 1*d*). Here we assume that the active rate of RhoA increases when the vertex angle satisfies Δ*θ*^*i*^ < −*c*_0_*θ*_0_ due to the cell polarization. The coupling strength between the local protrusion dynamics of a vertex *i* and its active RhoA concentration is represented by the coefficient $\kappa_{b}^{+}$. We set the value of $\kappa_{b}^{+}$ equal to the coefficients $\alpha_{\rho }$ and $\beta_{\rho }$ to ensure that the magnitude of the third term during a strong protrusion becomes comparable to the first two terms to suppress an unbounded protrusion event, thereby ensuring numerical stability.

Similarly, Rac1 activation rate $A_{R}^{i}$ at the *i*th vertex also consists of three terms,

A 2 $$A_{R}^{i}=\frac{\alpha_{R}{R_{a}^{i}}^{3}}{\left({R_{0}}^{3}+{R_{a}^{i}}^{3}\right)}+\frac{\beta_{R}{\rho_{0}}^{3}}{\left({\rho_{0}}^{3}+{\rho_{a}^{i}}^{3}\right)}+e^{H(\Delta \theta^{i}-c_{0}\theta_{0})\frac{\Delta \theta^{i}}{\theta_{0}}}K_{b}^{+},$$

where $K_{b}^{+}$ governs the coupling strength between the local contractile dynamics and active RhoA concentration at vertex *i*. We take the value of the $K_{b}^{+}$ to be equal to the coefficients *α*_*R*_ and *β*_*R*_. We assume that the active rate of Rac1 soars once the increase of the vertex angle exceeds the threshold *c*_0_*θ*_0_ due to the cell contraction. Coupling Rac1 and RhoA to the mechanical deformations enables recurrent polarization and the recovery of cell deformations. Specifically, an excessive increase in *θ*^*i*^ at the retracting cell rear builds up $R_{a}^{i}$ to increase actin polymerization and thus reverses the retraction at the end of each migration step. A large decrease in *θ*^*i*^ at the protruding cell front boosts *ρ*_*a*_ that increases actomyosin contraction to resist further protrusion. This two-way chemomechanical coupling in our model is essential for the successive cell polarization and apolarization events while exerting minimal influence on average cell spreading area and migration speed.

**A.2. Analytical derivation of the soft substrate force and deformation**

We have derived an analytical expression for the substrate force *F*_sub_ at a single FA site to explain the influence of viscosity on a soft substrate. The derivation focuses on isolated motor-clutch dynamics that can also be numerically solved by equations (2.2)–(2.6) (electronic supplementary material, figure S15A). For a steady-state solution, we neglect the time-dependence of the variables *N*_*c*_, *N*_*m*_, *F*_*p*_ by setting $N_{c}=N_{c}^{0}$, $N_{m}=N_{m}^{0}$, *F*_*p*_ = 0, which are otherwise determined by the chemo-mechanical dynamics at the cellular scale. It is important to note that our goal is not to replicate the substrate force at each FA site of the proposed whole-cell model but to decipher the mechanism of the non-monotonic profile between the viscosity and substrate force on soft substrates. Such motor-clutch analyses that ignore the details of the global dynamics prove to be effective in understanding the influence of matrix mechanics on traction forces [17,23,45,46]. To that end, the analytical derivations presented are aimed at mathematically demonstrating the effect of viscosity.

The molecular clutches bind between the actin filaments and the substrate with a reference association rate $k_{\mathrm{on}}^{0}$, or equivalently at a timescale $\tau_{\mathrm{on}}^{0}\equiv 1/k_{\mathrm{on}}^{0}$. At any instant, the number of the associated clutches is $n_{c}\equiv PN_{c}^{0}$. The substrate force builds up at an FA site with the actin filaments moving toward the cell nucleus with the retrograde speed $\left(V_{r}\equiv {\dot{x}}_{r}\right)$. The total restoring force of the bounded clutches is balanced by the substrate deformation. By first considering an elastic substrate, we have *K*_*e*_*x*_sub_ = *n*_*c*_*K*_*c*_(*x*_*r*_ − *x*_sub_). Since *n*_*c*_ ≫ 1, it is easy to show that the clutch deformation (*x*_*r*_ − *x*_sub_) is insignificant compared with the substrate deformation *x*_sub_, or *x*_*r*_ ≈ *x*_sub_ [45]. Therefore, we have the force–velocity relation in an alternative form,

A 3 $${\dot{x}}_{\mathrm{sub}}\approx V_{0}\left(1-\frac{F_{\mathrm{sub}}}{N_{m}^{0}f_{m}}\right).$$

On an elastic substrate, equation (A 3) only contains one unknown *x*_sub_ and can be solved with the initial condition *x*_sub_|_*t*=0_ = 0,

A 4 $$x_{\mathrm{sub}}=\frac{N_{m}^{0}f_{m}}{K_{e}}(1-e^{-t/\tau_{l}}).$$

The substrate force is thus given by

A 5 $$F_{\mathrm{sub}}=N_{m}^{0}f_{m}(1-e^{-t/\tau_{l}}).$$

The timescale $\tau_{l}=\frac{N_{m}^{0}f_{m}}{V_{0}K_{e}}$ sets the substrate deformation rate and can approximate the lifetime of a binding–unbinding cycle in the FA dynamics [28]. By comparing the substrate deformation timescale *τ*_*l*_ with the binding timescale $\tau_{\mathrm{on}}^{0}$, we defined a threshold stiffness $K_{0}=N_{m}f_{m}k_{\mathrm{on}}^{0}/V_{0}$ (table 2). On soft substrates with *K*_*e*_/*K*_0_ < 1, the clutch tension builds up slowly, and a long lifetime $\left(\tau_{l}>\tau_{\mathrm{on}}^{0}\right)$ allows for the association of a large number of clutches, i.e. $n_{c}∼N_{c}^{0}$. On stiff substrates, the traction force increases rapidly, and a short lifetime $\left(\tau_{l}<\tau_{\mathrm{on}}^{0}\right)$ limits the clutch association. Consequently, the force of a single engaged clutch increases significantly (*f* > *f*_cr_ in equation (2.3)), triggering adhesion reinforcement.

By describing a viscoelastic substrate by the SLS model, the substrate force–displacement relation is given by

A 6 $$(K_{e}+K_{a})\gamma {\dot{x}}_{\mathrm{sub}}+K_{a}K_{e}x_{\mathrm{sub}}=K_{a}F_{\mathrm{sub}}+\gamma {\dot{F}}_{\mathrm{sub}}.$$

Since equation (A 3) still holds on viscoelastic substrates as long as the instantaneous stiffness is below the threshold, i.e. *K*_*e*_ + *K*_*a*_ < *K*_0_, we substitute equation (A 3) into equation (A 6) to eliminate *F*_sub_, which gives

A 7 $$\tau_{r}{\ddot{x}}_{\mathrm{sub}}+\left[1+\frac{\tau_{r}}{\tau_{l}}\left(1+\frac{K_{a}}{K_{e}}\right)\right]{\dot{x}}_{\mathrm{sub}}+\frac{1}{\tau_{l}}x_{\mathrm{sub}}=V_{0},$$

where *τ*_*r*_ ≡ *γ*/*K*_*a*_ defines the relaxation timescale. With the initial conditions *x*_sub_ = 0 and *F*_sub_ = 0 at *t* = 0, the solution of the substrate force is obtained as

A 8 $$\begin{array}{r} F_{\mathrm{sub}}=N_{m}^{0}f_{m}\left(1-\frac{{\dot{x}}_{\mathrm{sub}}}{V_{0}}\right)=N_{m}^{0}f_{m}[1+\left(\frac{\tau_{2}-\tau_{l}}{\tau_{1}-\tau_{2}}\right)\\ \;\times \exp\left(-\frac{t}{\tau_{1}}\right)+\left(\frac{\tau_{l}-\tau_{1}}{\tau_{1}-\tau_{2}}\right)\exp\left(-\frac{t}{\tau_{2}}\right)], \end{array}$$

where *τ*_1_ and *τ*_2_ represent two characteristic times with *τ*_1_ ≡ *τ*_*l*_ + *τ*_*r*_(1 + (*K*_*a*_/*K*_*e*_)), and *τ*_2_ ≈ *τ*_*r*_*τ*_*l*_/*τ*_1_. For soft substrates with a low equilibrium stiffness *K*_*e*_ ≪ *K*_*a*_ (i.e. *K*_*a*_/*K*_*e*_ ≫ 1), we have *τ*_2_/*τ*_*l*_ = 1/((1 + *K*_*a*_/*K*_*e*_) + *τ*_*l*_/*τ*_*r*_) ≪ 1 and *τ*_2_/*τ*_1_ = 1/(2(1 + *K*_*a*_/*K*_*e*_) + *τ*_*l*_/*τ*_*r*_ + *τ*_*r*_/*τ*_*l*_(1 + *K*_*a*_/*K*_*e*_)^2^) ≪ 1. We can thus simplify the equation as

A 9 $$\begin{array}{r} F_{\mathrm{sub}}=N_{m}^{0}f_{m}[1-\frac{\tau_{l}}{\tau_{l}+\tau_{r}\left(1+\frac{K_{a}}{K_{e}}\right)}\exp\left(-\frac{t}{\tau_{1}}\right)\\ \;-\frac{\tau_{r}\left(1+\frac{K_{a}}{K_{e}}\right)}{\tau_{l}+\tau_{r}\left(1+\frac{K_{a}}{K_{e}}\right)}\exp\left(-\frac{t}{\tau_{2}}\right)]. \end{array}$$

The solutions have good agreement with the numerical results prior to the clutch rupturing at *t* ≈ *τ*_*l*_ (electronic supplementary material, figure S15B). Equation (A 9) can be simplified if we focus on two extreme cases. For a substrate with a short relaxation time $\left(\tau_{r}\leq \tau_{\mathrm{on}}^{0}\right)$, the long duration of the binding–unbinding cycles allows us to neglect the influence of short characteristic time *τ*_2_, which gives

A 10 $$F_{\mathrm{sub}}\approx N_{m}^{0}f_{m}\left[1-\frac{\tau_{l}}{\tau_{1}}\exp\left(-\frac{t}{\tau_{1}}\right)\right],\;\text{when}\;\tau_{r}\leq \tau_{\mathrm{on}}^{0}.$$

By contrast, a very viscous substrate corresponds to a long relaxation timescale *τ*_*r*_ ≥ *τ*_*l*_, making the contribution of the second term in the square bracket negligible, i.e.

A 11 $$F_{\mathrm{sub}}\approx N_{m}^{0}f_{m}\left[1-\exp\left(-\frac{t}{\tau_{2}}\right)\right],\;\text{when}\;\tau_{r}\geq \tau_{l},$$

where $\tau_{2}\approx N_{m}^{0}f_{m}/(K_{a}+K_{e})$, same as the characteristic time on an elastic substrate (equation (A5)) with the stiffness *K*_*a*_ + *K*_*e*_.

With these analytical solutions, we can calculate the average substrate force of binding/unbinding cycle by ${\bar{F}}_{\mathrm{sub}}=\int_{0}^{\tau_{l}}F_{\mathrm{sub}}dt/\tau_{l}$. When $\tau_{r}\leq \tau_{\mathrm{on}}^{0}$, the clutch system preserves the lifetime on an elastic substrate, i.e. $\tau_{l}\approx N_{m}^{0}f_{m}/V_{0}K_{e}$. By contrast, a long relaxation time means that the substrate deforms like an elastic substrate with a stiffness *K*_*a*_ + *K*_*e*_. As a result, the shortened lifetime is comparable to the characteristic timescale *τ*_2_ of the substrate deformation when *τ*_*r*_ > *τ*_*l*_. Thus, the average substrate force in two limits is given by

A 12 $${\left\langle F_{\mathrm{sub}}\right\rangle }_{\tau_{l}}\approx \left\{ \begin{array}{cc} N_{m}^{0}f_{m}\exp\left[\frac{-\tau_{l}}{\tau_{l}+\tau_{r}(1+(K_{a}/K_{e}))}\right], & \text{if}\;\tau_{r}\leq \tau_{\mathrm{on}}^{0}\\ N_{m}^{0}f_{m}\exp\left(-1\right), & \text{if}\;\tau_{r}\geq \tau_{l}. \end{array}\right.$$

Although the average substrate traction force is difficult to solve for when $\tau_{\mathrm{on}}^{0}<\tau_{r}\leq \tau_{l}$, our numerical results demonstrate a fast decrease of the clutch lifetime when $\tau_{r}>\tau_{\mathrm{on}}^{0}$, leading to a quick drop of the average substrate force (electronic supplementary material, figure S15C). Taken together, the average traction force must vary in a biphasic fashion with increasing viscosity and achieve a maximum when $\tau_{r}\approx \tau_{\mathrm{on}}^{0}$ (electronic supplementary material, figure S15D).

The above derivations approximate the dynamics of the traction force at a single FA site with *N*_*c*_ ≈ *N*_*m*_. We next briefly explain the impact of viscosity on the net traction force, which is the traction difference between the cell front and rear. On soft substrates with *K*_*a*_ + *K*_*e*_ < *K*_0_, the traction force decreases with increasing viscosity beyond $\gamma >\tau_{\mathrm{on}}^{0}K_{a}$ due to the reduced FA lifetime (electronic supplementary material, figure S15C). Consequently, the net traction force must also vary biphasically with increasing viscosity. Owing to the regulation of Rho GTPase molecules, the front of the cell has a higher number of clutches than motors ($N_{c}^{i}>N_{m}^{i}$), reducing the tension ($f_{c}^{i}$) shared by each bond and thus prolonging the clutch lifetime (electronic supplementary material, figure S15E). The extended FA lifetime preserves the high traction force at the front of the cell even when $\tau_{r}>\tau_{\mathrm{on}}^{0}$ (electronic supplementary material, figure S15F). This explains why the viscosity that maximizes the net traction force is slightly higher than the viscosity that produces the maximum traction force at the rear FA sites.

**A.3. Estimation of individual clutch force under adhesion reinforcement**

On very stiff substrates, the rapid building of tension $f_{c}^{i}$ on an individual clutches contributes to a large average bond force far beyond the threshold force (i.e. ${\left\langle f_{a}\right\rangle }_{\tau_{l}}\gg f_{\mathrm{cr}}$), giving rise to very strong adhesion reinforcement effect. The association rate can thus be approximated by $k_{\mathrm{on}}^{i}\approx k_{\mathrm{on}}^{0}\exp\left[\zeta ({\left\langle f_{a}\right\rangle }_{\tau_{l}}-f_{\mathrm{cr}})\right]$. For an engaged clutch with well-developed tension $f_{c}^{i}$, the clutch disassociation rate is mainly dominated by effect of the slip bond, i.e. $k_{\mathrm{off}}^{i}\approx k_{r0}\exp(\;f_{c}^{i}/f_{r0})$. For the saturation of the bounded clutches on very stiff substrates, we must have d*P*^*i*^/d*t* = 0 and $k_{\mathrm{on}}^{i}=(1-P^{i})/P^{i})k_{\mathrm{off}}^{i}\approx ck_{\mathrm{off}}^{i}$ (equation (2.4)), where *c* denotes a constant. Therefore, we know that the bond force sustained by an individual clutch can be related to the adhesion reinforcement coefficient by $f_{c}^{i}/f_{r0}\propto \zeta ({\left\langle f_{a}\right\rangle }_{\tau_{l}}-f_{\mathrm{cr}})$. Typically, the maximum of ${\left\langle f_{a}\right\rangle }_{\tau_{l}}$ in our numerical analyses is around 10–12 pN. We can thus further obtain that $f_{c}^{i}/f_{r0}\propto 8\zeta \;\mathrm{pN}$.
